# Prospective study of dietary patterns and colorectal cancer among Singapore Chinese

**DOI:** 10.1038/sj.bjc.6604678

**Published:** 2008-09-23

**Authors:** L M Butler, R Wang, W-P Koh, M C Yu

**Affiliations:** 1 Division of Epidemiology, Department of Public Health Sciences, University of California-Davis, Davis, CA 95616, USA; 2 The Masonic Cancer Center, University of Minnesota, Minneapolis, MN 55455, USA; 3 Department of Community, Occupational and Family Medicine, National University of Singapore, Singapore 117597, Singapore

**Keywords:** colorectal cancer, diet, dietary patterns, principal components analysis, prospective cohort, Singapore Chinese

## Abstract

An influence of Western diet and lifestyle factors observed among Singapore Chinese may contribute to the population's marked rise in colorectal cancer incidence over the past two decades. Thus far, however, there is little evidence for individual nutrients and foods as major contributing factors in this population. We evaluated whether patterns of food intake were associated with colorectal cancer in a population-based cohort of 61,321 Singapore Chinese that was established in 1993–98. Two dietary patterns, meat–dim sum and vegetable–fruit–soy, were previously identified by principal components analysis using baseline dietary data from a validated 165-item food frequency questionnaire. As of 31 December 2005, 961 incident colorectal cancer cases were diagnosed. Proportional hazards regression was used to calculate adjusted hazard ratios. Using nearly 10 years of follow-up data, we observed no association with either the meat–dim sum or vegetable–fruit–soy pattern for colorectal cancer. In conclusion, neither individual nutrients or foods nor dietary patterns appear to explain the rise in colorectal cancer among Singapore Chinese population.

Coinciding with gaining political independence in 1965, Singapore, a country of 4 million people, mostly of Chinese ethnicity experienced tremendous economic growth (Ministry of Trade and Industry, 2005). Along with the rise in industrialisation and urbanisation there was a shift in the major causes of death from infectious diseases to ‘lifestyle’ diseases common in Western societies, such as cancer, cardiovascular disease, and stroke (Ministry of Health, 2006). This shift was also evident with cancer trends, where cancers associated with infection, such as stomach, nasopharynx, liver, have been decreasing in incidence, whereas cancers of the prostate, breast, and colorectum have been on the rise (Seow *et al*, 2004). Colorectal cancer, in particular, has increased by over 120% among men and women in the past two decades (Wong and Eu, 2007), approaching the incidence rates seen among US Chinese (McCracken *et al*, 2007).

Using data from a prospective cohort of 63 257 Singapore Chinese, we have investigated diet by analysing associations with individual nutrients and foods thought to be aetiologically relevant to colorectal cancer (World Cancer Research Fund/American Institute for Cancer Research, 2007). While valuable, this strategy probably did not capture the complex interactions between individual nutrients and their correlations with other dietary (Randall *et al*, 1990; Kant *et al*, 1991), lifestyle (Slattery *et al*, 1999; Maskarinec *et al*, 2000), and sociodemographic patterns (Gex-Fabry *et al*, 1988; Lv and Cason, 2004) that may confound associations with colorectal cancer. This is one plausible explanation for the more consistent epidemiologic findings with dietary patterns and colorectal cancer, compared to those with nutrients and foods. For example, several prospective studies support a positive association with dietary patterns characterised by red meat, potatoes, sweets, and fried foods (Fung *et al*, 2003; Dixon *et al*, 2004; Kesse *et al*, 2006; Flood *et al*, 2008). However, few data address dietary patterns among Chinese populations in relation to colorectal cancer (Seow *et al*, 2002), and to our knowledge, none have evaluated these associations in a prospective study.

## Materials and methods

The design of the Singapore Chinese Health Study has been previously described (Yuan *et al*, 2003). The cohort consisted of 63 257 men and women recruited between April 1993 and December 1998, from permanent residents or citizens of Singapore aged 45–74 years, and who resided in government-built housing estates (86% of the Singapore population resided in such facilities). We restricted the study to individuals belonging to the two major dialect groups of Chinese in Singapore: the Hokkiens and the Cantonese. Participants completed a baseline in-person interview that elicited information on diet, demographics, current physical activity, reproductive history (women only), occupational exposure, and medical history. For these analyses, we used data from the 61 321 individuals who did not have a history of cancer diagnosis at baseline. The Institutional Review Boards at the University of Minnesota and the National University of Singapore have approved this study.

Incident colorectal cancer cases and deaths among cohort members were identified by record linkage of the cohort database with the population-based Singapore Cancer Registry and the Singapore Registry of Births and Deaths. The nationwide cancer registry has operated since 1968 and is comprehensive in its ascertainment (Parkin *et al*, 2003). To date, only 17 cases were known to be lost to follow-up due to migration out of Singapore. As of 31 December 2005 (an average of 9.8 years of follow-up), 961 cohort participants developed invasive colorectal cancer (591 colon, 370 rectal/rectosigmoid cancers).

At baseline, a 165-item quantitative food frequency questionnaire, developed for and validated in this population, was administered to assess usual diet over the past year (Hankin *et al*, 2001). Principal components analysis among the baseline cohort (*N*=63 257) was used to identify dietary patterns from the food frequency responses, details of which have been reported (Butler *et al*, 2006). Briefly, the number of components retained for orthogonal rotation was based primarily on examination of scree plots and factor interpretability, but eigenvalues (>1.0) and percent variance explained were also considered (Hatcher, 1994). For each component, a score was computed as a linear composite of the foods with meaningful loading scores (e.g., ⩾0.30). Scores were calculated by taking the unweighted sum of standardised frequencies of intake for each food associated with the pattern, then dividing them into quartiles based on the distribution of the baseline cohort. We labelled the two distinct dietary patterns: ‘vegetable–fruit–soy’ and ‘meat–dim sum’ (See Appendix Table A1). Briefly, the vegetable–fruit–soy pattern was characterised by vegetable, fruit, and soyfood intake; of the 32 foods included in the pattern, 23 were vegetables, five were soyfood items, and four were fruit items. The meat–dim sum pattern contained 31 food items, predominantly chicken, pork, fish, rice and noodle dishes, and preserved foods. Eleven of the nineteen dim sum or snack items on the questionnaire were included in this pattern. No food or beverage items overlapped between the two patterns.

### Statistical analysis

Person-years of follow-up were counted from the date of recruitment to the date of diagnosis of colorectal cancer, death, migration, or 31 December 2005, whichever occurred first. Proportional hazards regression methods were used to examine the associations between dietary exposure levels and colorectal cancer risk, measured by hazard ratios (HRs) and their corresponding 95% confidence intervals (CIs) (Cox, 1972). Study participants were grouped into quartiles based on the distribution among the entire cohort. The linear trend tests for dietary pattern – cancer associations were based on ordinal values of the quartiles (0, 1, 2, 3). In all analyses, we adjusted for the following potential confounders: sex, age at baseline interview (years), year of interview (1993–1995, 1996–1998), dialect group (Cantonese, Hokkien), the level of education (no formal education, primary school, secondary school, or higher), cigarette smoking (‘heavy’=started to smoke before the age of 15 years and smoked ⩾13 cigarettes per day, ‘light’=all non-heavy smokers, never) (Tsong *et al*, 2007), alcohol consumption (nondrinker, <7 drinks per week, ⩾7 drinks per week) (Tsong *et al*, 2007), body mass index (<20, 20–23.9, 24–27.9, ⩾28 m kg^−2^), familial history of colorectal cancer (no, yes first-degree relative), diabetes at baseline (no, yes) (Seow *et al*, 2006), any weekly physical activity (no, yes).

## Results

The Singapore Chinese diet consists primarily of mixed dishes that are generally high in refined carbohydrates (e.g., noodles and white rice), soyfoods, and green leafy vegetables, and contain relatively small quantities of meats, such as chicken and pork. Indicative of a population in transition from low to high socioeconomic status, we observed a wide distribution of intake for traditional (e.g., fish and soy) and non-traditional (e.g., red meat and dairy) foods (Table 1). With the exception of a positive association with marine n-3 PUFA and alcohol intake, none of the nutrients or foods was associated with colorectal cancer (Table 1).

Individuals among the fourth quartile of the meat–dim sum pattern were more likely to be male, have higher education, report any weekly physical activity, be a heavy smoker, drink alcohol, and consume more saturated fat, compared to individuals in the first quartile (Table 2). Similar trends by education level and physical activity were observed between fourth and first quartiles of fruit–vegetable–soy pattern intake. In contrast, individuals in the fourth fruit–vegetable–soy quartile were less likely to be heavy smokers, and more likely to consume marine n-3 and total n-6 PUFAs.

We observed no association between quartiles of the meat–dim sum or vegetable–fruit–soy dietary patterns and risk of colorectal cancer, overall or by stage of disease (Table 3). With the meat–dim sum pattern, there were no differences in HRs for fourth *vs* first quartile by subsite (HR=0.95, 95% CI: 0.72–1.25 for colon; HR=1.04, 95% CI: 0.73–1.49 for rectum), or by sex (HR=0.95, 95% CI: 0.72–1.26 for men; HR=0.96, 95% CI: 0.68–1.37 for women). Similarly, there was no association with the vegetable–fruit–soy pattern, regardless of subsite or sex (data not shown). No effect modification of either dietary pattern was observed by body mass index, physical activity, education level, or baseline diabetes (data not shown).

## Discussion

Using nearly 10 years of follow-up data from our Singapore Chinese cohort, we observed no association with dietary patterns for colorectal cancer. Our findings contribute to the few prospective studies of dietary patterns and cancer risk among Asian populations (Kim *et al*, 2005; Cui *et al*, 2007). Our finding of no association with a dietary pattern characterised primarily by vegetable intake is consistent with most prospective findings for similar dietary patterns among Japanese (Kim *et al*, 2005), US (Fung *et al*, 2003; Wu *et al*, 2004), and Western European (Dixon *et al*, 2004) populations. However, our finding of no association with our meat-based pattern was not consistent with most previous studies.

Our meat–dim sum pattern was similar to the ‘Western’ dietary pattern, previously characterised by red and processed meats, sweets and desserts, French fries, refined grains (Fung *et al*, 2003). Positive associations have been reported among most (Fung *et al*, 2003; Dixon *et al*, 2004; Kesse *et al*, 2006; Flood *et al*, 2008), but not all (Terry *et al*, 2001; Wu *et al*, 2004) US and Western European cohorts. Among the Japanese cohort, no association was observed with the Western pattern and colorectal cancer overall, but a positive association with a non-statistically significant trend was observed for colon cancer among women (Kim *et al*, 2005).

Perhaps there is more convincing evidence for a positive association with a Western dietary pattern among non-Asian populations, because the pattern is more strongly correlated with other colorectal cancer risk factors, such as obesity and physical inactivity. Differences in meat cooking methods between Western and Asian populations may be another reason for the discrepancy. For example, in the US consuming grilled meat is a major source of exposure to the colorectal mutagens, heterocyclic amines (Bogen and Keating, 2001), whereas these are at non-detectable levels among Chinese populations, where stir frying is the preferred meat cooking method (Turesky *et al*, 2007).

These and previous findings from the Singapore Chinese cohort do not support hypotheses that dietary factors, whether as single nutrients, foods, or as dietary patterns, are major contributors to the recent rise in colorectal cancer risk in this population. Individual dietary factors with evidence for a role in colorectal cancer include red meat, preserved red meat, and alcohol as risk factors; and dietary fiber, garlic, dairy products, and calcium as preventive factors (World Cancer Research Fund/American Institute for Cancer Research, 2007). Of these factors, only alcohol intake was associated with colorectal cancer in our data (Tsong *et al*, 2007). Neither alcohol nor the other dietary risk factors that we have identified in our data, such as saturated and marine n-3 polyunsaturated fatty acids (Butler *et al*, in press) and green tea (Sun *et al*, 2007), are likely to explain the recent colorectal cancer trends among Singapore Chinese population.

As an alternative hypothesis, we propose that the rise in type 2 diabetes mellitus (T2D) prevalence in Singapore (Cheah *et al*, 1985; Ministry of Health, 1998) is a major contributing factor to the parallel rise in colorectal cancer risk. We have reported a modest positive association between T2D and colorectal cancer (Seow *et al*, 2006). We have also observed a monotonic increase in risk of developing T2D across the spectrum of body mass index, with a moderate association for those among the second decile (HR=1.70; 95% CI=1.20–2.41, for 18.8–20.2 *vs* 11.6–18.8 kg m^−2^) up to a HR of 7.80 (95% CI: 5.80–10.48) for the top decile (Odegaard *et al*, 2006). Possible underlying biologic mechanisms by which insulin resistance may cause colorectal cancer include stimulating proliferation and reducing apoptosis in colon cells, inducing change in cell signalling pathways, such as protein kinase-C and mitogen-activated protein kinase, and alterering the insulin growth factor system, which is responsible for cell growth and differentiation (reviewed by Gunter and Leitzmann, 2006).

The limitations of principal components analysis include the subjective nature of determining the number of patterns, labelling the patterns, and interpreting these patterns (Martinez *et al*, 1998). However, we conducted sensitivity analyses and found a high degree of internal consistency and reproducibility with our patterns (Butler *et al*, 2006). These limitations are far outweighed by the strengths of our study, including the use of an food frequency questionnaire that was developed for and validated in our population (Hankin *et al*, 2001). In addition, the prospective design of our study reduced the opportunity for differential dietary recall to bias our findings.

In conclusion, neither individual nutrients or foods nor dietary patterns appear to be the underlying explanation for the rise in colorectal cancer among Singapore Chinese. Population trends, in addition to a modest association with T2D in our cohort, as well as a strong underlying biologic mechanism, all suggest that factors associated with insulin resistance, such as visceral adiposity and physical inactivity, may be appropriate targets for reducing colorectal cancer incidence in the Singapore Chinese population.

## Figures and Tables

**Table 1:** Single nutrient and food associations with colorectal cancer

	**5th percentile, median, 95th percentile**		**HR (95% CI)** a	***P* for trend**
	**Cases (*N*=961)**	**Noncases (*N*=60 360)**	**4th *vs* 1st quartile**	
Total kcal	799, 1463, 2538	821, 1460, 2593	0.97 (0.80–1.18)	0.77
				
*Nutrients, per 1000 kcal*				
Dietary fiber (g)	4.4, 7.7, 13	4.6, 7.9, 13	0.98 (0.81–1.19)	0.78
Starch (g)	73, 107, 151	68, 104, 146	1.09 (0.90–1.31)	0.56
Folate (*μ*g)	56, 93, 144	58, 96, 158	0.86 (0.71–1.04)	0.32
Calcium (mg)	129, 232, 476	136, 240, 496	0.91 (0.76–1.09)	0.19
Vitamin D (IU)	16, 58, 141	18, 58, 142	1.09 (0.92–1.31)	0.20
Selenium (*μ*g)	50, 66, 86	49, 65, 84	1.14 (0.95–1.37)	0.14
Total soy isoflavones (mg)	1.6, 9.2, 26	1.9, 9.8, 29	0.95 (0.79–1.13)	0.81
Total fat (g)	14, 24, 33	25, 16, 34	0.98 (0.81–1.19)	0.84
Saturated fat (g)	4.4, 8.4, 13	4.9, 8.8, 13	0.97 (0.80–1.17)	0.68
Animal fat (g)	3.3, 8.0, 14	3.3, 8.2, 14	1.13 (0.94–1.35)	0.35
Marine n-3 PUFA (g)	0.06, 0.18, 0.35	0.06, 0.18, 0.35	1.21 (1.01–1.45)	0.03
n-6 PUFA (g)	2.2, 4.1, 7.6	2.3, 4.2, 8.0	0.91 (0.75–1.10)	0.79
				
*Foods, g per day*				
Red meat	5.0, 24, 74	4.4, 25, 76	1.01 (0.82–1.26)	0.60
Preserved meat	0, 1.2, 8.5	0, 1.3, 10	1.16 (0.95–1.41)	0.10
Fish	14, 51, 111	14, 51, 114	1.17 (0.96–1.43)	0.07
Vegetables	33, 89, 208	37, 98, 228	0.98 (0.79–1.21)	0.72
Fruit	4.0, 151, 472	12, 166, 514	0.89 (0.72–1.09)	0.46
Soy foods	14, 83, 254	16, 88, 286	0.95 (0.78–1.16)	1.0
Dairy products	0.30, 18, 272	0.38, 20, 279	0.98 (0.82–1.17)	0.88
Alcohol (drinks per week)	0, 0, 10	0, 0, 5.2	1.58 (1.23–2.04)^b^	0.001

CI=confidence interval.

a All hazard ratios (HRs) were adjusted for age at interview (year), sex, dialect group (Cantonese, Hokkien), interview year (1993–1995, 1996–1998), diabetes at baseline (no, yes), smoking history (never, ‘heavy’ or ⩾13 cigarettes per day starting age <15 years, ‘light’ or non-heavy smokers), body mass index (<20, 20–23.9, 24–27.9, ⩾28 m kg^−2^), alcohol intake (0, <7, ⩾7 drinks per week), education (no formal education, primary school, secondary school, or higher), any weekly physical activity (no, yes), first-degree relative diagnosed with colorectal cancer (no, yes), and total daily energy intake (kcal).

b HR for ⩾7 drinks per week *vs* nondrinker.

**Table 2:** Baseline characteristics by dietary pattern quartiles (Q)

	**Meat – dim sum**		**Vegetable – fruit – soy**	
	**Q1**	**Q4**	**Q1**	**Q4**
Person years (No.)	153 028	148 434	156 658	144 410
Median age (interquartile range) years	59 (13)	53 (11)	57 (14)	54 (12)
Male (%)	31.2	59.6	47.9	43.4
Hokkien dialect group (%)	56.0	52.9	58.9	50.1
				
*Highest level of education (%)*				
No formal education	37.8	17.3	34.2	21.5
Primary education	41.8	45.6	44.1	43.9
Secondary education or higher	20.4	37.1	21.7	34.6
				
*Body mass index, kg per m* ^*2*^ *(%)*				
<20.0	15.2	15.5	15.7	15.3
20.0–24.0	56.7	52.1	56.1	52.2
24.1–28.0	21.4	24.8	21.4	25.3
>28.0	6.8	7.6	6.8	7.3
Any weekly physical activity (% yes)	29.9	37.6	26.4	38.7
				
*Smoking index (%)*				
Never	77.4	60.7	62.0	73.7
Light	20.0	34.2	32.9	23.4
Heavy	2.7	5.1	5.0	2.9
				
*Alcohol consumption (%)*				
Nondrinkers	90.9	68.9	81.5	79.4
<7 drinks per week	7.1	22.6	12.6	16.1
⩾7 drinks per week	2.0	8.5	5.9	4.4
				
*Green tea intake (%)*				
None	67.2	50.9	69.7	48.6
Monthly	10.1	12.4	10.3	12.3
Weekly	12.5	22.2	10.9	23.3
Daily	10.2	14.4	9.1	15.9
Diabetes (% yes)	10.4	7.7	9.6	8.4
				
*Median daily intake (interquartile range)*				
Energy, kcal	1165 (472)	1947 (780)	1211 (576)	1800 (772)
Saturated fat, g per 1000 kcal	7.3 (3.2)	10.4 (3.2)	8.0 (3.4)	9.7 (3.6)
Polyunsaturated fat (PUFA), g per 1000 kcal	4.4 (2.5)	5.0 (1.9)	4.1 (1.9)	5.5 (2.7)
Marine n-3 PUFA, g per 1000 kcal	0.18 (0.13)	0.18 (0.10)	0.16 (0.12)	0.19 (0.12)
Total n-6 PUFA, g per 1000 kcal	3.9 (2.4)	4.4 (1.8)	3.6 (1.8)	4.8 (2.5)

**Table 3:** Hazard ratios (HR) for dietary pattern quartiles (Q) in relation to colorectal cancer by stage of disease

	**Q1**	**Q2**	**Q3**	**Q4**
*Meat–dim sum pattern*				
*Colorectal cancer*				
Cases, *N*	277	250	225	209
HR^a^	1.0	1.01	0.98	0.97
95% CI		0.85–1.21	0.81–1.18	0.78–1.20
				
*Localised disease*				
Cases, *N*	96	94	107	79
HR^a^	1.0	1.09	1.30	0.99
95% CI		0.81–1.45	0.97–1.74	0.69–1.41
				
*Advanced disease*				
Cases, *N*	161	140	107	119
HR^a^	1.0	0.98	0.81	0.99
95% CI		0.78–1.24	0.62–1.05	0.74–1.32
				
*Vegetable–fruit–soy pattern*				
*Colorectal cancer*				
Cases, *N*	283	243	224	211
HR^a^	1.0	1.00	1.01	1.02
95% CI		0.84–1.19	0.84–1.21	0.83–1.24
				
*Localised disease*				
Cases, *N*	101	88	98	89
HR^a^	1.0	1.01	1.22	1.16
95% CI		0.75–1.34	0.91–1.62	0.84–1.59
				
*Advanced disease*				
Cases, *N*	161	143	114	109
HR^a^	1.0	1.04	0.92	0.95
95% CI		0.82–1.30	0.71–1.18	0.73–1.25

CI=confidence interval.

a All HRs were adjusted for age at interview (year), sex, dialect group (Cantonese, Hokkien), interview year (1993–1995, 1996–1998), diabetes at baseline (no, yes), smoking history (never, ‘heavy’ or ⩾13 cigarettes per day starting age <15 years, ‘light’ or non-heavy smokers), body mass index (<20, 20–23.9, 24–27.9, ⩾28 m kg^−2^), alcohol intake (0, <7, ⩾7 drinks per week), education (no formal education, primary school, secondary school, or higher), any weekly physical activity (no, yes), first-degree relative diagnosed with colorectal cancer (no, yes), and total daily energy intake (kcal).

**Table A1:** Factor loadings for foods associated with each dietary pattern: Singapore Chinese Health Study (Butler *et al*, 2006)

**Vegetable–fruit–soy**			**Meat–dim sum**		
**Food item**	**Food type** a	**Loading factor ( × 100)**	**Food item**	**Food type** a	**Loading factor ( × 100)**
Cauliflower	V	55	Siew mai	DS, M	46
Broccoli	V	50	Other steamed snack	DS, M	44
Carrots	V	49	Gravy noodle	M, St	43
Green beans/peas	V	48	Chicken rice	M, St	42
Other plain tofu in soups, mixed dishes or alone	S	46	Otar otar (spiced fish paste)	DS	41
Tung goo	V	43	Pork satay	M	40
Tomatoes	V	43	Chicken, mutton curry	M	39
Corn	V	43	Glutinous rice dumpling	DS, St	39
Yin choi, po choi	V	43	Steamed meat bao	DS, M	39
Kai lan	V	42	Popiah	DS, M	38
Head lettuce, Chinese lettuce	V	42	Ngor hiang	DS, M	38
Gum jum, dried fungus	V	42	Chinese rojak	DS, V	37
Tou gay, tai tau nga	V	41	Puffs, such as curry or bean	DS, V	37
Pak choy, siew pak choy	V	41	Chicken satay	M	37
Choi sum	V	41	Roasted duck or goose	M	37
White potatoes	V	40	Coconut rice dishes	St	36
Other tau kwa	S	40	Roti prata	St	36
Fu kua, mo qua	V	39	Other pig organs	M	36
Watercress	V	39	Preserved eggs	O	35
Head cabbage, wong nga pak	V	38	Deep fried chicken	M	35
Celery	V	37	Coconut desserts	DS	34
Foojook vegetarian meats	S	36	Other flavoured rice, such as duck or char siew	St	34
Kai choi	V	36	Curry rice	St	34
Apple	F	35	Deep-fried snack, such as jian dui, fried prawn or fish ball, etc.	DS	33
Cucumber	V	34	Dry noodle dish, such as fishball, chicken or wanton mee	M, St	33
Yong tau foo	S	33	Belly pork	M	33
Other tau pok in soups, mixed dishes, or alone	S	33	Lup chong	M	33
Other dark green leaves	V	32	Other fried noodle	St	32
Papaya	F	31	Luncheon meat	M	31
Pear	F	31	Fried rice	St	30
Honeydew	F	31	Squid	M	30
Gee choi	V	30			
% variance explained		7.3			7.2
Coefficient *α*		0.81			0.76

a V=vegetable, S=soyfood, F=fruit, M=meat dish (includes fish/shellfish), DS=dim sum/snack dish, St=high-starch item (e.g., rice dish, noodle dish, bread), O=other.

## Appendix A

Table A1
